# 1680. Characterization of Ethanol Lock Use and Line Infections in Pediatric Patients with Intestinal Failure Requiring Central Venous Access

**DOI:** 10.1093/ofid/ofad500.1513

**Published:** 2023-11-27

**Authors:** Karen Davidge, Elizabeth Mroczka, Chelsey Ivy, Jerod Nagel

**Affiliations:** University of Michigan Health, Ann Arbor, Michigan; University of Michigan, Troy, Michigan; University of Michigan College of Pharmacy, Ann Arbor, Michigan; Michigan Medicine, Ann Arbor, Michigan

## Abstract

**Background:**

Pediatric patients with intestinal failure often require central venous line access for administration of parenteral nutrition and/or fluids, which leads to increased risk for central line associated-bloodstream infections (CLABSI). Ethanol lock therapy (ELT) has been used to reduce the risk of CLABSI in this patient population, but recent significant increases in the price of ethanol have prompted healthcare systems to explore cost-saving measures such as decreasing ELT frequency from daily to thrice weekly (TIW) or using antibiotic lock therapy as an alternative to ELT. The objective of this study was to compare the incidence and microbiology of CLABSI in pediatric patients with intestinal failure who received different lock therapies.

**Methods:**

This was a single-center, retrospective, descriptive study that included pediatric patients who were followed by the Michigan Medicine Children’s Intestinal Rehabilitation Program between July 1, 2020 and June 30, 2022. Data were collected via the electronic health record. Incidence of CLABSI and microbiology were compared based on the type of lock therapy patients received (daily ELT, TIW ELT, or no ELT).

**Results:**

A total of 46 patients were analyzed in this study, including 13 on daily ELT, 23 on TIW ELT, and 10 on no ELT. During the study period, 69% (9/13) of patients on daily ELT, 35% (8/23) of patients on TIW ELT, and 60% (6/10) of patients on no ELT had at least one CLABSI (for comparison between daily and TIW ELT, p = 0.0819). Of 75 episodes of CLABSI identified during the study period, the most commonly isolated pathogens were *Staphylococcus epidermidis* (n = 33), *S. aureus* (n = 19), *S. hominis* (n = 17), and *Escherichia coli* (n = 8). Almost half (44%, 33/75) of CLABSI reported were polymicrobial (see Table 1 for comparison of microbiology by type of lock therapy).

**Table 1:**
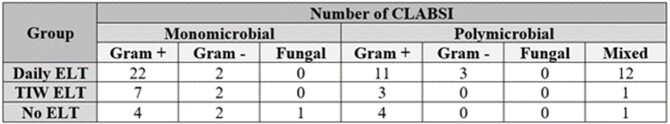
Number of monomicrobial vs. polymicrobial CLABSI by Gram stain results and type of lock therapy.

**Conclusion:**

There was no statistically significant difference in the incidence of CLABSI between pediatric patients with intestinal failure who received TIW ELT and those who received either daily ELT or no ELT. TIW ELT appears to be an effective and cost-efficient option compared to daily ELT in this patient population.

**Disclosures:**

**All Authors**: No reported disclosures

